# lncRNA PSORS1C3 is regulated by glucocorticoids and fine-tunes OCT4 expression in non-pluripotent cells

**DOI:** 10.1038/s41598-019-44827-7

**Published:** 2019-06-10

**Authors:** Fatemeh Mirzadeh Azad, Mahshid Malakootian, Seyed Javad Mowla

**Affiliations:** 1 Department of Molecular Genetics, Faculty of Biological Sciences, Tarbiat Modares University, Tehran, Iran; 2 Cardiogenetic Research Center, Rajaie Cardiovascular Medical and Research Center, Iran University of Medical Sciences, Tehran, Iran

**Keywords:** Mutagenesis, Gene expression, Gene regulation, Long non-coding RNAs, Transcriptional regulatory elements

## Abstract

OCT4 is a transcription factor known for its regulatory roles in stemness, tumorigenesis and stress response. Considering its versatile functions, expression of OCT4 is regulated at different levels. PSORS1C3, a long non-coding RNA overlapped with OCT4, has a putative association with immune mediated diseases; however, its exact functions remained to be elucidated. Here, we demonstrated that PSORS1C3 is regulated by glucocorticoids (GC), has two endogenously active promoters, promoter 0 and 1, and two sets of transcripts, short and long variants. According to our findings, PSORS1C3 promoters behaved differently during neural differentiation of NT2 cells and glucocorticoid receptor (GR) activation. In both processes the expression pattern of short variants differed from that of long variants and was similar to OCT4 expression. Furthermore, our data revealed that PSORS1C3’s promoter 0 could act as an enhancer for OCT4 in non-pluripotent cells, where its deletion caused a significant decrease in OCT4 expression. Meanwhile, during GR activation promoter 0 functioned as a negative regulator and alleviated transcription induction of OCT4 after GC treatment. Altogether, our work clarified the structure and regulation of PSORS1C3, explained its relation to immune-related disease through GR signaling and introduced it as a novel fine-tuner of OCT4 expression in non-pluripotent cells.

## Introduction

OCT4, also named POU5F1, is the main regulator of pluripotency in stem cells^1^. It also has a part in tumorigenesis, cellular stress response, and hence is linked to various diseases from cancer to autoimmune disorders^2–4^. OCT4 possesses several isoforms with different functions at both protein and transcript levels, and their expressions are regulated through different mechanisms and at various biological processes^5,6^.

One of the main stages of gene expression regulation occurs at transcription level. Using different promoters, enhancers or silencers, and transcription start sites, genes differentially respond to different biological signals^7^. Aside from transcription initiation, OCT4 expression is also regulated during splicing and post transcription by non-coding RNAs^8^.

Long non-coding RNAs (lncRNAs) are non-protein coding transcripts with a size of more than 200 nucleotides. They are capable of regulating gene expression with different mechanisms such as acting as guiding molecules, scaffold and RNA or protein decoy or distributer. Apart from their transcripts, the genomic elements residing in lncRNAs locus, like their promoters, could exert regulatory roles on their neighboring or even distant genes^9–11^.

*PSORS1C3*, a lncRNA located in close vicinity to *OCT4*, was first discovered in a linkage analysis on psoriasis^12^. We have recently reported the existence of several novel exons and splice variants as well as two potential promoters for *PSORS1C3*^13^. Aside from linkage association of PSORS1C3 to psoriasis and other immune mediated diseases such as acute anterior uveitis^14^, recently Murphy *et al*. in their methylation analysis of brain samples from suicide committed depressed patients suggested that PSORS1C3 was related to Major Depressive Disorder^15^. However, scrutinizing the location of their proposed differentially methylated region (DMR) and comparing it with our previous findings^13^, indicated that the DMR was overlapping the promoter of POU5F1 novel variant, OCT4C, and not PSORS1C3. Nevertheless, the exact molecular function of PSORS1C3 or its probable regulatory effect on *OCT4* gene was not identified.

Here by analyzing PSORS1C3 promoter activity, we identified network of transcription factors that could bind to the region and tried to understand the regulation behind *PSORS1C3* expression. We also knocked out PSORS1C3 promoter and assessed its effect on *OCT4* expression.

## Results

### Transcription activity of PSORS1C3 locus

**Figure 1:**
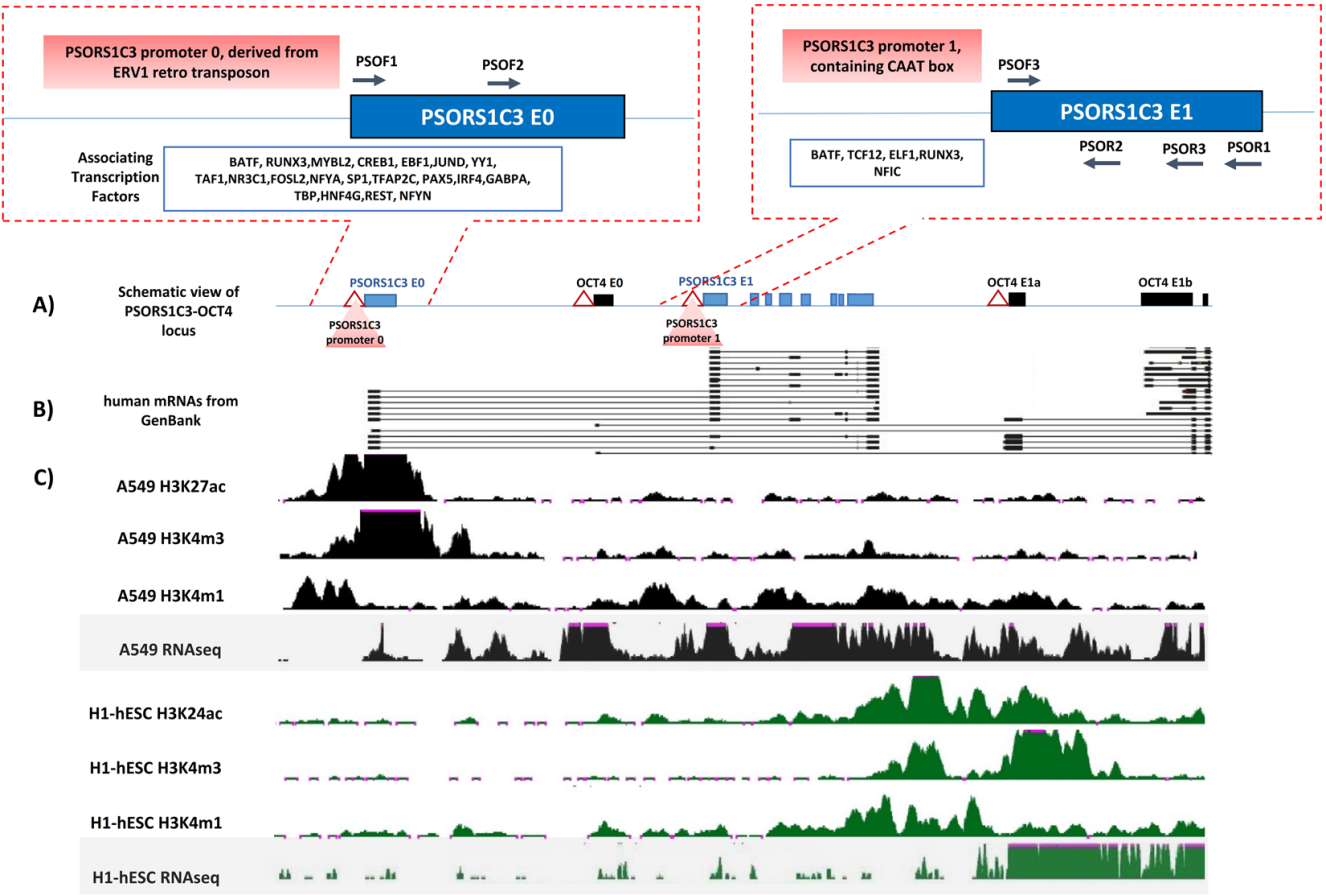
A visualization of ENCODE data on different histone modifications and transcription in PSORS1C3-OCT4 locus. (**A**) Schematic view of PSORS1C3-OCT4 locus, containing novel spliced exons (filed boxes), regions with promoter activity (triangles) and their associated transcription factors. (**B**) Group of PSORS1C3 short and long transcripts submitted to GenBank. (**C**) Histone modifications and transcription status of the locus according to ENCODE data in A549 and H1 cells.

Reviewing ENCODE data demonstrated that the expression and chromatin modifications along PSORS1C3-OCT4 locus were different in stem and non-stem cells (Fig. 1A,B). As it can be observed in Fig. 1C, in A549 cells the active marks (H3K27ac, H3K4me3 and H3K4me1) were mostly concentrated at PSORS1C3 promoter upstream of exon 0 (hereafter promoter 0), but the transcription activity was detected throughout the locus (including OCT4). However, the chromatin active marks as well as transcription in H1 hESC was restricted to OCT4 immediate promoter and proximal enhancer regions. Moreover a weak transcriptional activity could be observed downstream of PSORS1C3 internal promoter (hereafter promoter 1).

According to ENCODE CHIPseq data, PSORS1C3 promoter 0 could interact with 22 different transcription factors (TF, Fig. 1A). Enrichment analysis showed that many of these TFs are in charge of transcription initiation or inhibition in immune reactions, response to extracellular stimuli and cellular stress. Among different TFs, NR3C1 or glucocorticoid receptor (GR) showed an intense signal around PSORS1C3 promoter 0 in CHIPseq data.

### PSORS1C3 promoter 0 was responsive to GR activation

**Figure 2:**
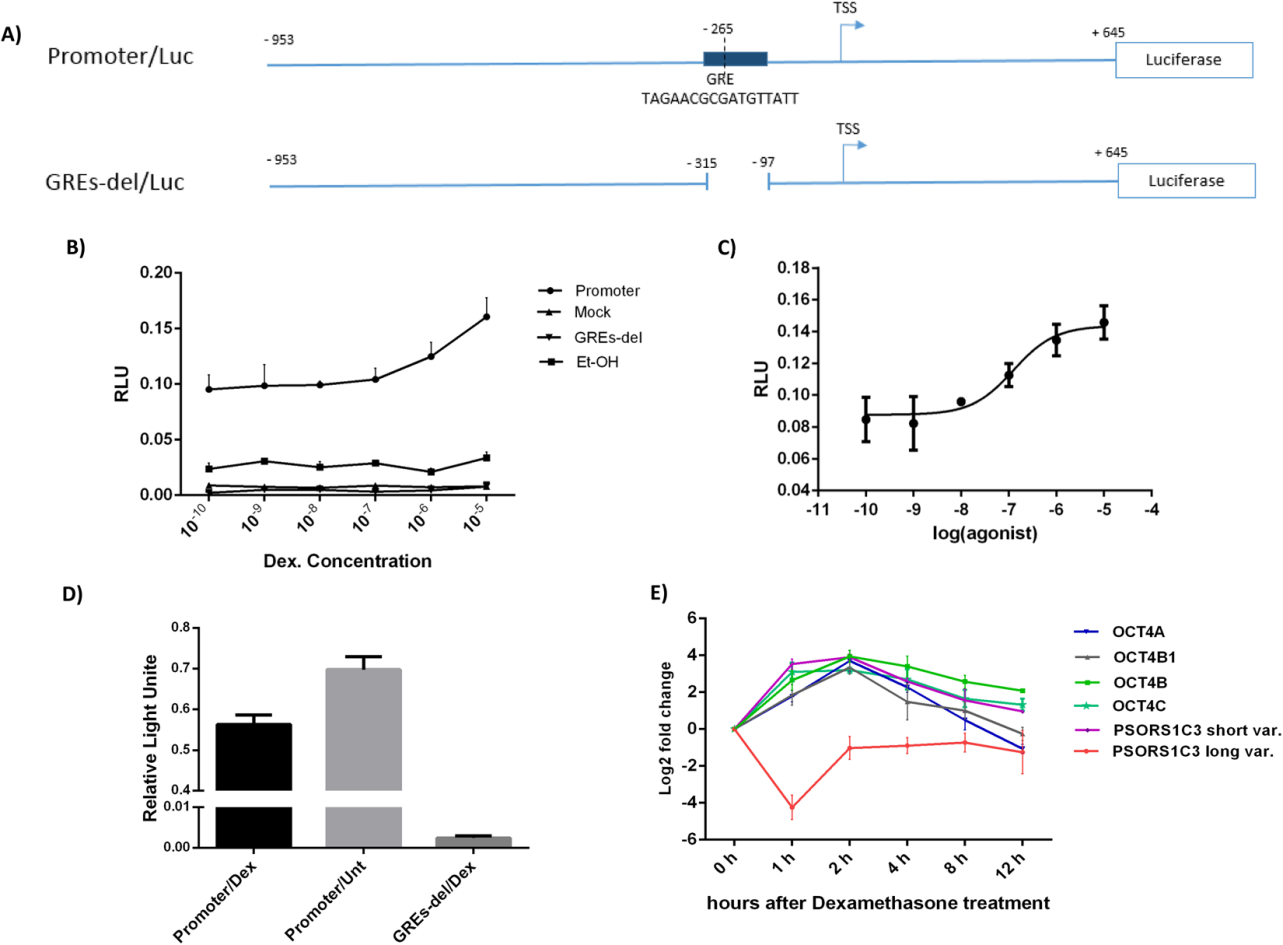
The effect of GR activation on PSORS1C3 and OCT4 expression levels. (**A**) Location of GRE in PSORS1C3 promoter 0 cloned into PGL4.14 vector. As a control we constructed a mutant promoter in which GRE was deleted by SOEing PCR. (**B**) Dual luciferase assay showed that the GRE in PSORS1C3 promoter was active and the promoter showed a dose dependent response to dexamethasone treatment. (**C**) The curve fit indicated that the EC50 value for dexamethasone is 100 nM. (**D**) The activity of PSORS1C3 promoter 0 in A549 cells decreased after treatment with 100 nM dexamethasone. (**E**) The inhibitory effect of GR activation on PSORS1C3 transcription was confirmed after treating A549 cells with DEX. The expression of PSORS1C3 long variants dropped significantly 1 h post treatment. Different isoforms of OCT4 gene showed a progressive up regulation after treatment followed by gradual down regulation. The expression alterations of PSORS1C3 short transcripts was similar to OCT4.

A dual luciferase assay on cells transfected with reporter vector containing PSORS1C3 promoter 0 (promoter-Luc), a mutant in which the GRE upstream of promoter 0 was deleted (GREdel-Luc) (Fig. 2A), and mock vector indicated a dose-dependent increase in luciferase activity, in HEK293T cells treated with different concentration of dexamethasone (DEX) and ethanol (used as DEX solvent, Fig. 2B). Dose curves fitting showed that EC50 value of DEX for inducing PSORS1C3 promoter 0 activity, was approximately 100 nM (Fig. 2C). In order to understand how the promoter 0 would behave in endogenous context, we transfected A549 cells (which were positive for PSORS1C3 expression) with promoter 0 construct and treated them with 100 nM DEX. The dual luciferase assay data revealed that DEX treatment caused a significant decrease (p value = 0.003) in PSORS1C3 promoter 0 activity, in cells transfected with promoter construct (Fig. 2D).

### Dexamethasone treatment affected PSORS1C3 and OCT4 transcripts levels

**Figure 3:**
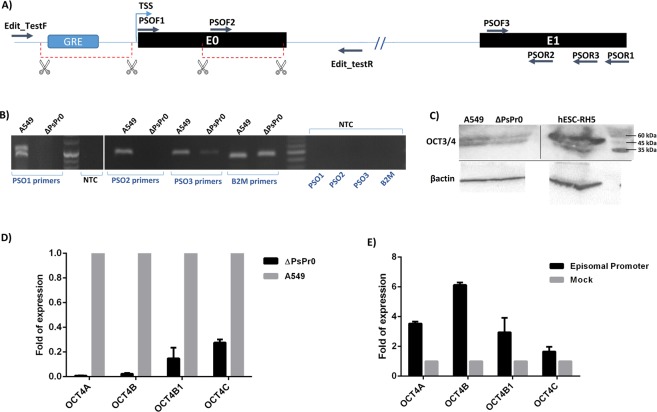
Effects of PSORS1C3 promoter knock out on OCT4 expression. (**A**) A schematic view of the deleted genomic region overlapping PSORS1C3 exon 0 and its upstream promoter using CRISPR/Cas9 system. The deleted region are shown by red border lines. The positioning of different sets of primers for detecting PSORS1C3 and gRNAs (scissors) are shown in the picture. (**B**) Suppression of E0 containing variants in edited cells (ΔPsPr0) was confirmed by RT-PCR using PSO1 and PSO2 primer sets. RT-PCR on ΔPsPr0 cells by PSO3 primer set could detect a weak expression for PSORS1C3 internal exons. Pictures of gels were cropped and juxtaposed and the full length gel pictures could be found in Supplementary Fig. 2. (**C**) Western blotting indicated the downregulation of Oct4 at protein level in ΔPsPr0 cells compared to un edited A549 cells. As it could be confer from the borders blot pictures were cropped and juxtaposed; Full length blots at different resolutions and exposures could be found in Supplementary Fig. 3. (**D**) Deletion of PSORS1C3 promoter 0 resulted in a significant decrease in several OCT4 isoforms. (**E**) Transfecting edited cells with the episomal vector containing the deleted DNA segment rescued OCT4 expression 48 hour after transfection.

In order to evaluate the possible effects of GR activation by dexamethasone on the expression of PSORS1C3 and its entangled gene, *OCT4*, A549 cells were treated with 100 nM dexamethasone and qRT-PCR was used to quantify the expression level of targeted genes. To evaluate PSORS1C3 expression in treated cells at different time points, we used a primer set specified to detect E0-E1 junction (PSO2, shown in Figs 1, 3) and a primer set corresponding to E1 (PSO3). Using the junctional primer set, we observed that one hour post-treatment the expression of PSORS1C3 E0 containing variants (long variants) dropped significantly (p value = 0.003), while qRT-PCR using non-junctional primer set showed an up-regulation for PSORS1C3 short variants (p value = 0.005, Fig. 2E).

The qRT-PCR results for OCT4 isoforms demonstrated that OCT4A, OCT4B, OCT4B1 and OCT4C transcripts levels elevated gradually after DEX treatment, with their peaks 2 hours after treatment, and declined then after (Fig. 2E). Their pattern of expression was similar to what we observed for PSORS1C3 during GR activation, when using the non-junctional primer set.

### PSORS1C3 has two endogenously active promoters and sets of transcripts

For suppressing PSORS1C3, we decided to knock out its most 5′ promoter region using CRISPR/Cas9 system. After confirming the genomic deletion of PSORS1C3 promoter 0, using PCR and DNA sequencing, we measured the expression of PSORS1C3 with different sets of primers (Fig. 3A). Using PSO1 and PSO2 primer sets, our RT-PCR data showed that the expression of long variants were eradicated in the knocked out cells (ΔPsPr0, Fig. 3B). When PSO3 primer set (corresponding to E1) was used, however, a weak expression of PSORS1C3 could be detected (Fig. 3B). The latter finding indicated that the PSORS1C3 promoter 1 could initiate transcription at a lower level and generate short variants.

### OCT4 variants were down-regulated in ΔPsPr0 cells

In order to assess any possible effects of PSORS1C3 promoter deletion on OCT4 expression, we used Western blotting and qRT-PCR to evaluate the expression of OCT4A protein and transcript along with three more isoforms of OCT4 namely OCT4B, OCT4B1 and OCT4C in ΔPsPr0 cells. Our data revealed that the expression of OCT4 isoforms were extremely decreased in the knocked out cells, compared to the unedited A549 cells. Our Western blotting data using polyclonal anti Oct3/4 antibody was also in line with the qRT-PCR results as a decrease in Oct4 protein level was observed in edited cells (Fig. 3C,D). Our data could imply that the deleted region might act as an enhancer for *OCT4* gene. To confirm the expression enhancing effect of the deleted area we transfected the ΔPsPr0 cells with a promoter-less vector containing the deleted sequence (promoter-luc). Our qPCR data confirmed that transfecting the edited cells with the episomal promoter sequence could rescue the OCT4 expression 48 hour post-transfection (Fig. 3E).

### GR activation in ΔPsPr0 cells caused a higher elevation in OCT4 isoform expression

**Figure 4:**
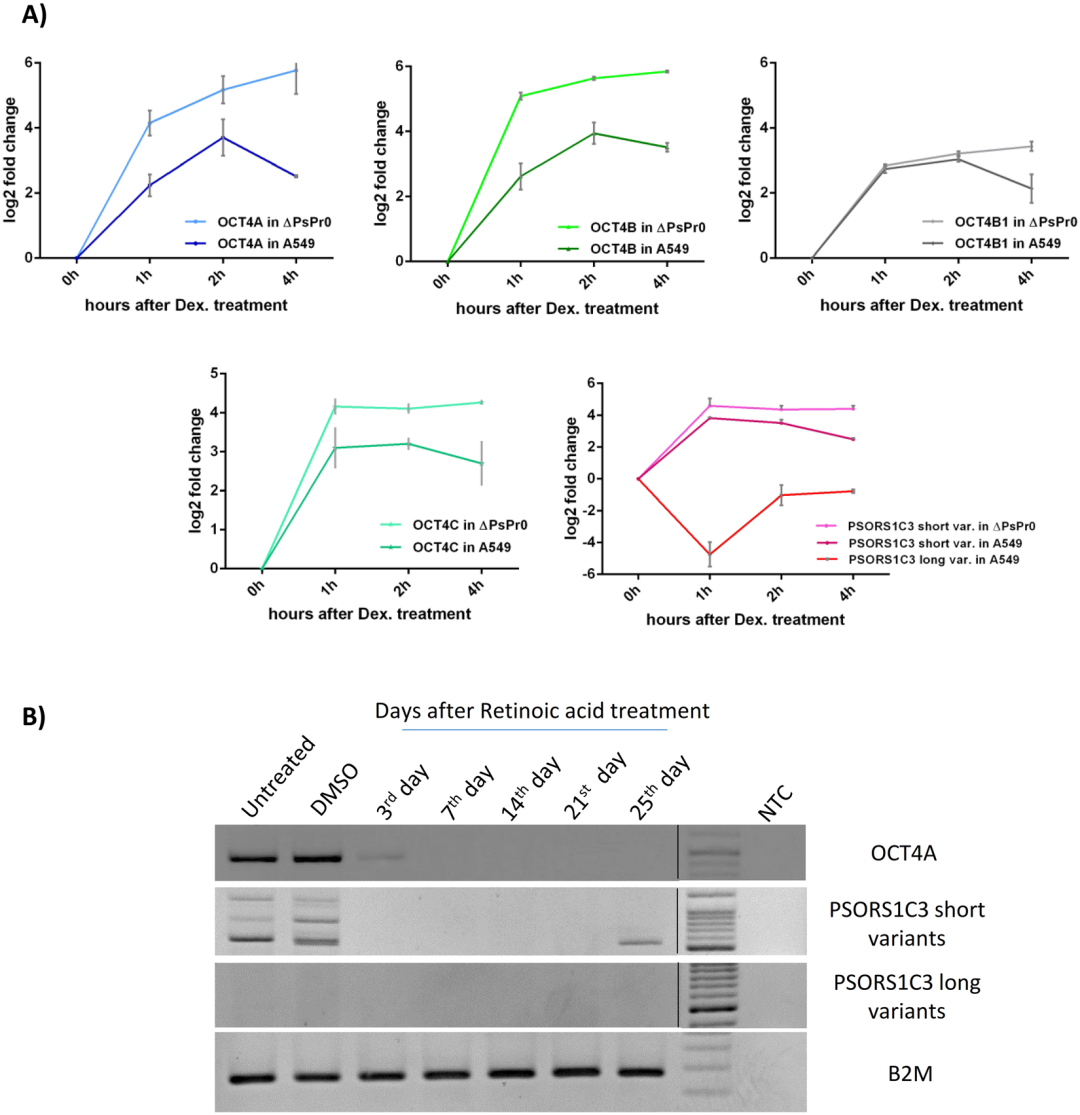
Different transcriptional behavior of PSORS1C3 promoters during GR activation and neural differentiation of NT2 cells. (**A**) The expression alterations of OCT4 isoforms and PSORS1C3 variants during GR activation in ΔPsPr0 and A549 cells. (**B**) Expression pattern of OCT4A (amplified by OCT4A-RTPCR primers), PSORS1C3 short and long variants (amplified with PSO5 and PSO4 primers sets respectively) in course of neural differentiation of NT2 cells. The expression alterations of PSORS1C3 short variants in course of neural differentiation of NT2 cells were more similar to expression pattern of OCT4A. RT-PCR samples for each transcript were run on a separate gel. Gel pictures were cropped and juxtaposed and full length gel could be seen in Supplementary Fig. 4.

We treated ΔPsPr0 cells with 100 nM DEX to examine whether deleting the GRE located in PSORS1C3 promoter upstream of E0 could affect the way OCT4 and PSORS1C3 short variants respond to GR activation. Surprisingly, we observed a much higher elevation in OCT4 expression level after DEX treatment in ΔPsPr0 cells, in comparison to the DEX treated A549 cells (Fig. 4A). Moreover, in A549 cells the expression of OCT4 isoforms started to decline 4 hours after treatment, whereas the same pattern was absent in the edited cells. PSORS1C3 short variants exhibited the same behavior in ΔPsPr0 cells after DEX treatment (Fig. 4A).

### Different behavior of PSORS1C3 variants during neural differentiation of NT2 cells

In order to investigate the possibility of a differential transcriptional activity of PSORS1C3 promoters 0 and 1, we evaluated the expression of PSORS1C3’s long and short sets of variants (using primer sets PSO4 and PSO5 respectively, Supplementary Table 1) as well as OCT4A during neural differentiation of NT2 cells. As it can be seen in Fig. 4B, PSORS1C3 short variants were expressed in undifferentiated NT2 cells, where their expression was lost during neural differentiation. In fact, their pattern of expression was similar to that observed for OCT4A variant. On the contrary, PSORS1C3 long variants were not detectable in NT2 cells, neither before nor after differentiation (Fig. 4B).

## Discussion

Transcriptional regulation is one of the main mechanisms for cells to control the expression of different genes^16^. OCT4 is a POU domain-containing transcription factor that regulates stemness, proliferation, stress response and cell cycle. The dosage of different isoforms of OCT4 transcripts and proteins is critical for the suitable regulatory outcome^17^. Therefore, cells use several strategies to fine-tune OCT4 expression and function, according to their needs^7,8,18^.

One of OCT4 neighboring gene is a lncRNA named PSORS1C3. In a recent report, we identified several spliced variants for PSORS1C3, starting from its canonical first exon. By discovering a novel starting exon for PSORS1C3, exon 0, we also extended its 5′ boundary, and discovered several long variants of PSORS1C3. In addition, we showed that different regions in PSORS1C3-OCT4 locus (upstream of PSORS1C3 exon 1 and exon 0) had the ability to initiate transcription in an episomal promoter reporter assay. However, the state of their endogenous activity was not clarified^13^.

In this study, we knocked out the most 5′ promoter (promoter 0) of PSORS1C3 to suppress its expression. After the deletion, a residual expression for PSORS1C3 internal exons was detected, thus PSORS1C3 promoter 1 was still active in edited cells. Regarding this findings, we concluded that both putative promoters were endogenously active and could generate different sets of transcripts. Furthermore, according to ENCODE data on chromatin accessibility and our expression analysis on edited cells, the internal promoter upstream of the PSORS1C3 exon 1 (promoter 1) was weaker than the LTR derived promoter upstream of exon 0.

Considering the difference in promoter’s type and their targeting transcription factors, it was expected that they would behave differently in diverse biological events^19–22^. Although CHIPseq data showed that GR had only one response element in OCT4-PSORS1C3 locus located upstream of PSORS1C3 promoter 0, but according to our results OCT4 and both PSORS1C3 transcription sets were differently responsive to GR activation. This observation could be explain by the fact that GR can enhance or repress the expression of its target genes directly or through second hand mediators^23,24^. Moreover, GR is a multitasking TF which regulates pro and anti-inflammatory pathways, proliferation and cellular stress all known to contribute to molecular pathology of immune mediated diseases like psoriasis^25–28^. Considering the regulatory role of OCT4 in proliferation and stress response, our result on positive effect of GR on expression level of OCT4 and PSORS1C3 short variants could be interpreted as a new regulatory path, in line with already known role of OCT4 in proliferation and stress response and proposing a novel role for PSORS1C3 short transcripts in these biological pathways.

The inhibitory effect of GR on PSORS1C3 promoter 0 and the expression of long variants highlights the possibility of its function as a pro-inflammatory factor. The occupancy of promoter 0 with TFs like SP1 which induces inflammatory signaling^29^ supports this idea. Our findings also indicated that the difference in expression response of PSORS1C3 short and long variants and the similarity of short variants behavior to OCT4 could be observed during neural differentiation of NT2 cells. The diversity in function of different isoforms of lncRNAs is reported before and is mainly due to formation of distinctive structural RNA domains in different splice variants^30,31^.

Knocking out PSORS1C3 promoter 0 led to a significant decrease in OCT4 isoforms expression. Therefore, it could be suggested that the promoter region also act as an enhancer for its neighboring gene *OCT4*. Several researches highlighted the importance of enhancer-like promoters (ePromoters) in regulating gene expression^32,33^. Recently, Diao *et al*. reported that the promoters of 17 protein-coding genes could act as an enhancer for OCT4 expression in hESC^34^. Aside from our experimental findings, mining ENCODE CHIPseq data demonstrated that PSORS1C3 promoter 0 shared some features of ePromoters. For example, it showed high level of occupancy by JUN, IRF and ATF/CREB family of transcription factors which are known to be associated with ePromoters, and modulate transcription of targeted gene in response to cellular stress^32,33^. It also could recruit YY1 protein, a factor involved in DNA looping^35^.

Despite our findings on the enhancing effect of PSORS1C3 promoter 0 on OCT4 expression, we observed that deleting the promoter area could intensify OCT4 upregulation during GR activation, and delay the rebound of its expression to the normal level. This negative effect could be explained by the fact that some DNA elements could function as both silencer and enhancer at different cells or biological states^36^. Moreover, some transcription factors that bind to PSORS1C3 promoter like BATF^37^, REST^38^, FOSL1^39^ and YY1^40^ could act as negative regulators of transcription. Thus, although the region could not completely lie into characteristics of a silencer elements, but by recruiting negative regulator of transcription could modulate and fine-tune the expression of OCT4. Moreover, according to ENCODE data, PSORS1C3 promoter 0 was only active in non-stem cells, therefore it could be suggested that its fine-tuning effect on OCT4 expression is restricted to somatic cancer cell lines in which the expression level of OCT4 transcripts are lower than that of stem cells.

Altogether, our work showed that PSORS1C3 is regulated by GC and could modulate OCT4 expression by acting as an enhancer as well as by recruiting negative regulators of transcription. Of course, it is needed to be further clarify the exact share of PSORS1C3 transcripts and genomic elements in its regulatory functions, but it is safe to suggest that PSORS1C3 could act as a fine tuner for OCT4 expression in non-pluripotent cells, in which the level of OCT4 is needed to be kept lower than that in stem cells.

## Methods

### Ethical statement

This research did not involve human participants or using human samples or tissues. It also did not include experimenting on live animals. The *in vitro* experiments on commercial cell lines and standard techniques of molecular genetics was approved as a PhD thesis proposal, by Tarbiat Modares Research Ethics Committee as well as research ethics committee of Rajaie Cardiovascular Medical and Research Center (reference number: RHC.AC.IR.REC.1396.61, year of approval: 2017).

### Bioinformatics analysis

We used UCSC genome browser to scan genomic area around PSORS1C3 first exon (coined exon 0, located at chr6:31,153,779-31,154,105(hg19)) for potential promoter activity. ENCODE^41^ CHIPseq data on chromatin active marks (H3K27Ac, H3K4m1 and H3K4m3) and DNaseq was used to detect the active area near transcription start site. Using ENCODE CHIPseq data for different transcription factors (TF), a list of TFs which bind to PSORS1C3 potential promoter was extracted. The TF binding sites was assigned using JASPAR software^42^. STRING software^43^ was used for building protein-protein interactions network and enrichment analysis.

### Cloning promoter constructs

After bioinformatic analysis, the potential promoter region for PSORS1C3 (−953 to +645, promoter 0) was amplified from genomic DNA using PFU (GeneAll, Korea) and specific primers (Supplementary Table 1) and was named Promoter-Luc. A mutant construct named GREdel-Luc, with deletion in GRE (−315 to −97) was built through SOEing PCR (Supplementary Table 1). The amplified constructs was directly cloned into PGL4.14 (Promega, USA) up stream of fire fly luciferase. The accuracy of cloned constructs were confirmed by sequencing (Macrogene, Korea).

### Cell culture

A549 and HEK293T cell lines were obtained from Iranian biological resource center (IBRC, Iran). NT2 cells were gifted by Dr. Andrews’s lab. A549 and NT2 Cells were cultured in high glucose Dulbecco’s Modified Eagle Medium (DMEM) and HEK293T cells were grown in DMED/F12 (Thermo Fisher Scientific, USA), supplied with 10% FBS (Thermo Fisher Scientific, USA), and 1% penicillin/streptomycin (Bio Basic, Canada) and incubated in 37 °C with 5% humified CO2. hESC-RH5 was obtained from Royan Institute for Stem Cell Biology and Technology (RI-SCBT, Iran) and was cultivated as described previously^44^.

For investigating the effects of dexamethasone treatment on the expression of targeted genes, cells were seeded in 24-well plates (SPL Life Sciences, South Korea). After complete adhesion, cells were treated with the EC50 concentration of dexamethasone and were lysed for RNA extraction at different time points.

### Promoter knock out using CRISPR/Cas9 system

In order to suppress PSORS1C3 expression, we decided to knock out its most 5′ promoter (promoter 0) using CRISPR/Cas9 system. Four different gRNAs were designed (Supplementary Table 1) to target the regulatory active area upstream of E0 using http://crispr.mit.edu/about. gRNAs where cloned into pX459 vector as described before^45^. gRNA containing vectors were transfected into A549 cells by Lipofectamin 2000 (Thermo Fisher Scientific, USA). Selection was done by treating cells 4 hours after transfection with Puromycin (Sigma-Aldrich, USA). Edited colonies were investigated for deletion by PCR using flanking primers (Edited-Test) and followed by DNA sequencing.

### Promoter reporter assay and EC50 calculation

HEK293T cells were seeded in 48-well plates (SPL Life Sciences, South Korea) and were transfected with Promoter-Luc, GREdel-Luc and mock vector using Lipofectamin 2000 (Thermo Fisher Scientific, USA), according to the manufacturer’s instruction. PGL4.74 vector (Promega, USA) containing Renilla luciferase was co-transfected with test vectors for further normalization and conferring transfection efficiency. Five hours after transfection, conditioned media was replaced with dexamethasone (Sigma-Aldrich, USA) containing media in different concentrations. To evaluate the probable false effect causing by ethanol, which was used as dexamethasone solvent, the same experiment was carried out by treating transfected cells with ethanol (Merck, Germany) containing media. Luciferase activity in the transfected cells was investigated 24 h after transfection by Dual-Luciferase® Reporter Assay System (Promega, USA) following the manufacturer’s protocol via FB12 tube luminometer (Titertek-Berthold, Germany).

After calculating the EC50 for DEX, A549 cells were transfected with promoter-Luc vector. 4 hours after transfection cells media was replaced with 100 nM DEX containing media. Luciferase activity was measured 24 hours post treatment, as mentioned before.

### Western blotting

Harvested cells were lysed with RIPA buffer (Sigma-Aldrich, USA) according to the manufacturer’s instruction. Protein concentration in lysate was qualified using Bradford assay. 30 µg of total protein for each sample was ran on SDS-PAGE and blotted on PVDF membrane (Sigma-Aldrich, USA) afterwards. After blocking with albumin, membrane was incubated with anti Oct3/4 antibody (sc-8629, Santa Cruz, USA) and anti β-actin antibody (ProSci, USA) overnight at 4 °C. Before visualizing the membrane with ECL kit (Lumigen, USA), it was incubated with HRP conjugated secondary antibody for 1 hour at RT.

### Neural differentiation of NT2 cells

For inducing neural differentiation in NT2 cells we used Peter Andrews protocol, as described before^46,47^. Briefly, cells were treated with 10–5 M all-trans-retinoic acid (RA) (Sigma-Aldrich, USA) for up to 21 days. Then, cells were passaged and re-cultured without adding retinoic acid to the medium. As a control group, NT2 cells were treated with 1% DMSO which was used as RA solvent.

### RNA extraction and qRTPCR

Cells were lysed for RNA extraction using TRIZOL (Thermo Fisher Scientific, USA) in different time points. Total RNA was extracted according to the manufacture instruction. 1 µg of RNA was first treated with DNase I (Thermo Fisher Scientific, USA) in order to eliminate DNA contamination and then was reversely transcribed using PrimeScript First Strand cDNA Synthesis Kit (TaKaRa, Japan). The expression of target gene was evaluated using BioFACT™ 2X Real-Time PCR Master Mix (BioFact, South Korea) through ABI StepOne real time PCR system (Thermo Fisher Scientific, USA). Primer sequence used for quantifying each target gene, could be found in Supplementary Table 1. Relative expression of target genes to beta 2-microglubolin (b2M) was calculated according to 2^−ΔΔCt^ method.

### Statistical analysis

All statistical analysis were done using GraphPad PRISM 6 software. Student t-test was used for investigating the significance of observed differences in gene expression levels and relative luciferase activity. All tests were done in 3 biological replicates and values were reported as mean ± standard deviation. P values below 0.05 was considered as statistically significant.

## Supplementary information

supplementary information
