# Supplementary material for: lncRNA PSORS1C3 is regulated by glucocorticoids and fine-tunes OCT4 expression in non-pluripotent cells
Source: Sci Rep. 2019 Jun 10;9:8370. doi: 10.1038/s41598-019-44827-7 (PMC6557835; doi:10.1038/s41598-019-44827-7)
Supplement: Supplementary file 1 — supplementary information [file 41598_2019_44827_MOESM1_ESM.pdf]

Supplementary information

lncRNA PSORS1C3 is regulated by glucocorticoids and fine-tunes OCT4 expression in non-pluripotent cells

Fatemeh Mirzadeh Azad[1], Mahshid Malakootian[2], Seyed Javad Mowla[1*]

[1]*Department of Molecular Genetics, Faculty of Biological Sciences, Tarbiat Modares University, Tehran, Iran*

[2]*Cardiogenetic Research Center, Rajaie Cardiovascular Medical and Research Center, Iran University of Medical Sciences, Tehran, Iran*

*Corresponding author:

Dr. Seyed Javad Mowla, Department of Molecular Genetics, Faculty of Biological Sciences, Tarbiat Modares University, Tehran, Iran. E-mail: sjmowla@modares.ac.ir; Tel: +9821-82883464; Fax: +9821-82884717

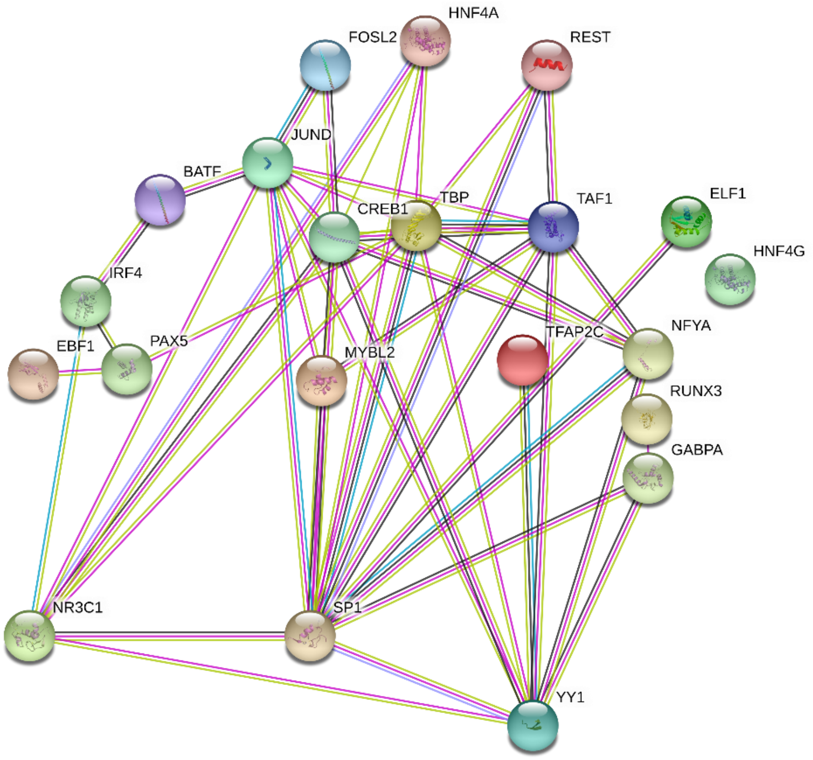

Supplementary figure 1: The protein – protein interaction network of transcription factors that bind to PSORS1C3 promoter 0 region. We used ENCODE CHIPseq data on different transcription factors (TFs) presented in UCSC genome browser to identify what TFs could bind to PSORS1C3 promoter 0 region (chr6:31,153,779-31,154,105(hg19)). According to ENCODE data, RUNX3, IRF4, BATF, SP1, PAX5, NR3C1, REST, TFAP2C, EBF1, YY1, JUND, HNF4A, CREB1, FOSL2, MYBL2, TAF1, GABPA, HNF4G, NFYN, NFYA, TBP and ELF1 could bind to PSORS1C3 promoter 0. STRING software was used for constructing protein-protein interactions network and enrichment analysis. [1]. Enrichment analysis showed that presented network of TFs could regulate different aspects of transcription initiation in cellular stress and responses to steroid hormones and extra cellular stimuli. SP1, YY1 and NR3C1 (glucocorticoid receptor) are three major nodes in the network.

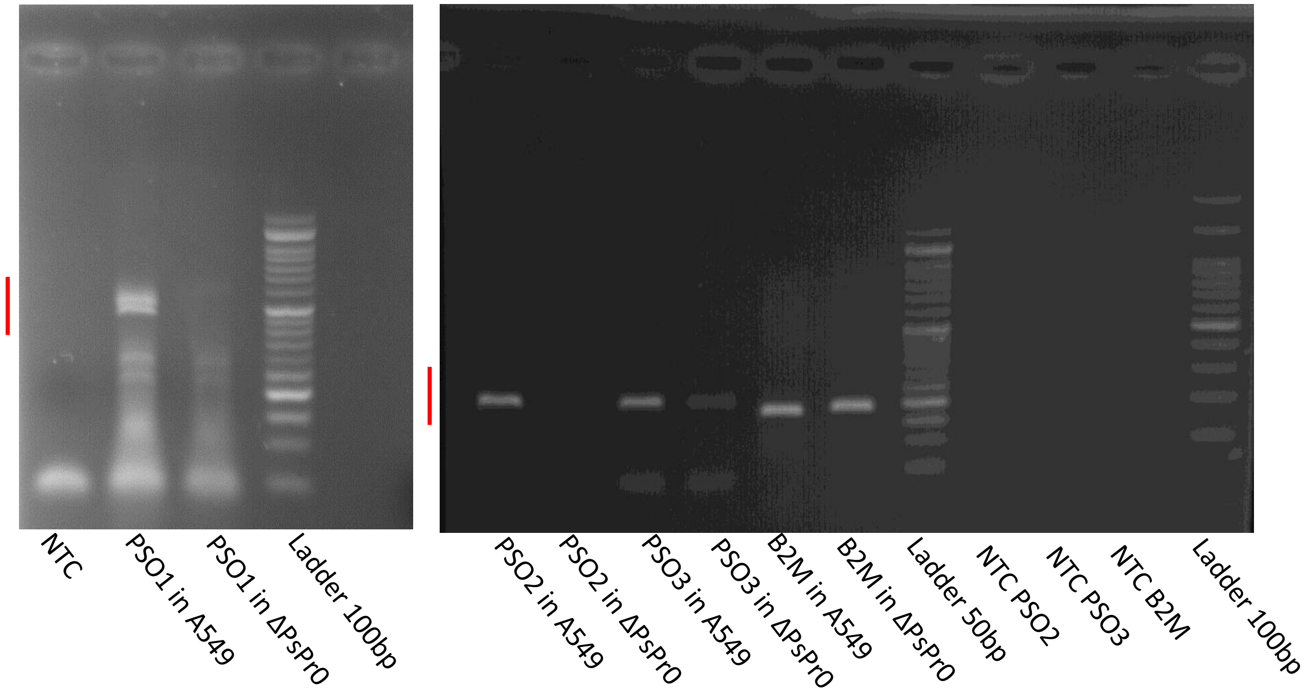

Supplementary figure 2: Full length gels used for constructing figure 3 B. Three different primer set, PSO1,PSO2,PSO3 were used to investigate PSORS1C3 expression after promoter 0 knock out in ΔPsPr0 cells and A549 cells as control group. Gel segments carrying relevant amplicon bands shown in figure 3 B, are specified with red lines at left side of the gels.

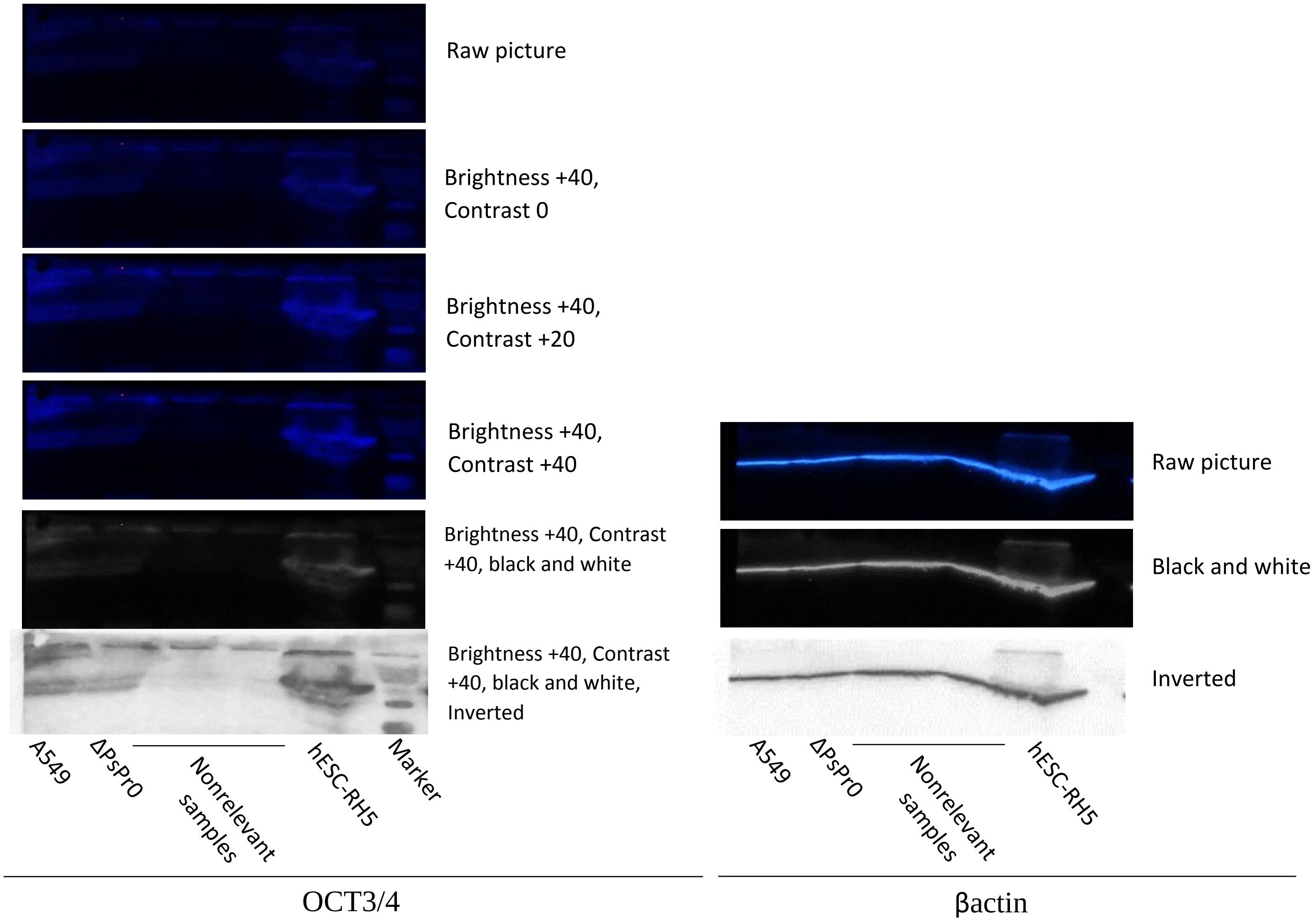

Supplementary figure 3: full length blots with different resolutions and exposures. Full length blots for OCT3/4 and βactin shown in figure 3 C, are presented with different exposures and resolutions. Blot areas which carry non-relevant samples and were omitted in the main figure, are specified with horizontal black line at the bottom of the last blot for each antibody.

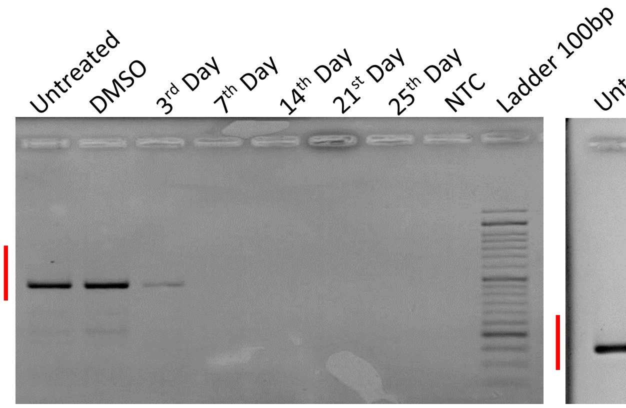

OCT4A (primer set:
OCT4A-RTPCR)

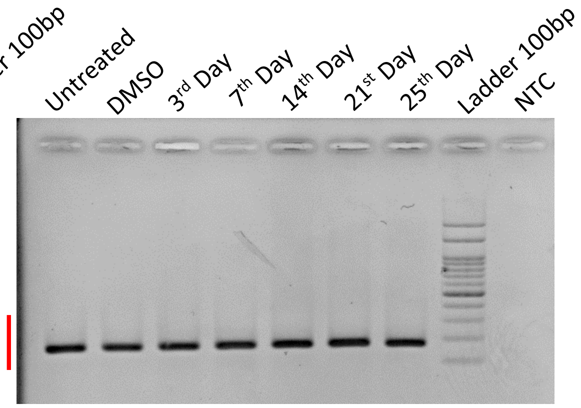

B2M

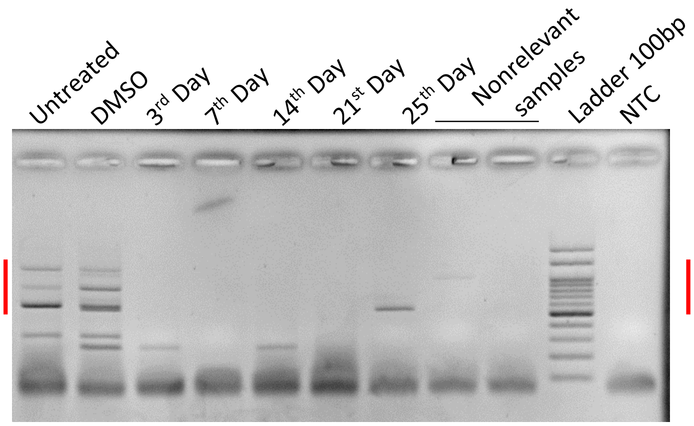

PSORS1C3 short variants
(primer set: PSO5)

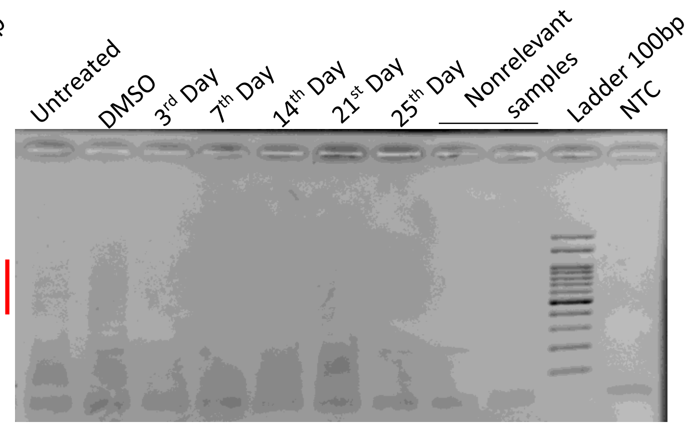

PSORS1C3 long variants
(primer set: PSO4)

Supplementary figure 4: full length gels used for constructing figure 4 B. Full length gels carrying amplicon bands for OCT4A, PSORS1C3 short variants, PSORS1C3 long variants and B2M which were used to build figure 4 B, are presented here. Primer sets used for amplifying each set of transcripts is specified under the gels. Gel segments carrying relevant amplicon bands shown in figure 3 B, are specified with red lines at left side of the gels.

Supplementary table 1: sequence of primers and oligos used in this research.

Useage	Name	Sequence 5´ to 3´	Amplicon size	Reference
Primers used for qRT-PCR	OCT4A	F:TCGCAAGCCCTCATTTC	113 bp	[2]
		R:CCATCACCTCCACCACCT		
	OCT4A-RTPCR	F:CTTCTCGCCCCCTCCAGGT	496 bp	[3]
		F:AAATAGAACCCCCAGGGTGAGC		
	OCT4B	F:AGACTATTCCTTGGGGCCACAC	244 bp	[3]
		R:GGCTGAATACCTTCCCAAATAGA		
	OCT4B1	F:AGACTATTCCTTGGGGCCACAC	272 bp	[3]
		R:CTTAGAGGGGAGATGCGGTCA		
	OCT4C	F:TGAGCGAGAAGCACGATCC	84 bp	
		R:GGAACGAACCGTCGC C		
	PSO1	F:GTTTTGTCTGGGGCTCGTC	497 bp	
		R:GCTCTGGCCTTCAGTCTGAGA		
	PSO2	F:CCAGAGCAGCACGTAGCAG	213 bp	
		R:CCCTCCTTGCAGCATCATAAG		
	PSO3	F:TGCTATGACTTCATTACTCTTTCCC	210 bp	
		R:GATGGTGACAAGGTGCTGGG		
	B2M	F:GGGTTTCATCCATCCGACATTG	167 bp	[3]
		R:TGGTTCACACGGCAGGCATAC		
	PSO4	F: GTTTTGTCTGGGGCTCGTC	*	[2]
		R: CTTCACACACACCTTTATTATTAC		
	PSO5	F:ACCCAGTCTCTGTGCTATGAC	†	[2]
		R:CTTCACACACACCTTTATTATTAC		
Primers used for investigating genomic edit	Edit-test	F: CCCCCATGTAATTCTTTCCACCA	1149 bp	
		R: TCTCCCTTAAATTCTTTCTTGAAA		
promoter-Luc	Soing 1	TCCCCTCTTCTAGGGTCTCTGTCTT	1588 bp	
	Soing 4	CTCGAGTCTCCCTTAAATTCTTTCTTGAAA		
GREdel-Luc	Soing 2	ACCAGGCCTGGGCCTGCCTGGCGAAATTGTGCATTCAGGGAGT	NA	
	Soing 3	GCCAGGCAGGCCCAGGCC	NA	
gRNA sequence for promoter 0	gRNA1	AACATCGCGTTCTAAGTCAT	NA	
	gRNA2	TCTACAACTCGGTGTCTGAA	NA	
	gRNA3	AGGTAACTGACGGACGGCCA	NA	
	gRNA4	CTTACCAAGTTTCAGATGTC	NA	

* Detects several long variants for PSORS1C3 starting from exon 0

† Detects PSORS1C3 short variants with different sizes starting from exon 1

References:

1. Von Mering, C., L.J. Jensen, B. Snel, S.D. Hooper, M. Krupp, M. Foglierini, et al., *STRING: known and predicted protein-protein associations, integrated and transferred across organisms.* Nucleic Acids Res, **33**(Database issue): p. D433-7 (2005).
2. Malakootian, M., F. Mirzadeh Azad, P. Naeli, M. Pakzad, Y. Fouani, E. Taheri Bajgan, et al., *Novel spliced variants of OCT4, OCT4C and OCT4C1, with distinct expression patterns and functions in pluripotent and tumor cell lines.* Eur J Cell Biol, **96**(4): p. 347-355 (2017).
3. Atlasi, Y., S.J. Mowla, S.A. Ziaee, P.J. Gokhale, and P.W. Andrews, *OCT4 spliced variants are differentially expressed in human pluripotent and nonpluripotent cells.* Stem Cells, **26**(12): p. 3068-74 (2008).
